# Risk Factors at Index Hospitalization Associated With Longer-term Mortality in Adult Sepsis Survivors

**DOI:** 10.1001/jamanetworkopen.2019.4900

**Published:** 2019-05-31

**Authors:** Manu Shankar-Hari, David A. Harrison, Paloma Ferrando-Vivas, Gordon D. Rubenfeld, Kathryn Rowan

**Affiliations:** 1 Intensive Care Unit Support Offices, St Thomas' Hospital, Guy's and St Thomas' NHS Foundation Trust, London, United Kingdom; 2 School of Immunology & Microbial Sciences, King’s College London, London, United Kingdom; 3 Intensive Care National Audit & Research Centre, London, United Kingdom; 4 Interdepartmental Division of Critical Care Medicine, Sunnybrook Health Sciences Centre, Toronto, Ontario, Canada

## Abstract

**Importance:**

Sepsis survivors, defined as adult patients who survived to hospital discharge following a critical care unit admission for sepsis, are at increased risk of long-term mortality. Identifying factors independently associated with long-term mortality, known during critical care admission for sepsis, could inform targeted strategies to reduce this risk.

**Objective:**

To assess, in adult sepsis survivors, factors independently associated with long-term mortality, known during their index critical care admission for sepsis, meeting Third International Consensus Definitions for Sepsis and Septic Shock criteria.

**Design, Setting, and Participants:**

This cohort study included a nationally representative sample of 94 748 adult sepsis survivors from 192 critical care units in England. Participants were identified from consecutive critical care admissions between April 1, 2009, and March 31, 2014, with survival status ascertained as of March 31, 2015. Statistical analyses were completed in June 2017.

**Exposures:**

Generic patient characteristics (age, sex, ethnicity, severe comorbidities [defined using the Acute Physiology and Chronic Health Evaluation II method], dependency, surgical status, and acute illness severity [scored using the Acute Physiology and Chronic Health Evaluation II acute physiology component]) and sepsis-specific patient characteristics (site of infection, number of organ dysfunctions, and septic shock status) known during index critical care admission for sepsis.

**Main Outcomes and Measures:**

Long-term mortality in adult sepsis survivors with maximum follow-up of 6 years. Adjusted hazard ratios (HRs) were estimated using Cox regression for both generic and sepsis-specific patient characteristics.

**Results:**

Sepsis survivors had a mean (SD) age of 61.3 (17.0) years, 43 584 (46.0%) were female, and 86 056 (90.8%) were white. A total of 46.3% had respiratory site of infection. By 1 year from hospital discharge, 15% of sepsis survivors had died, with 6% to 8% dying per year over the subsequent 5 years. Age, sex, race/ethnicity, severe comorbidities, dependency, nonsurgical status, and site of infection were independently associated with long-term mortality. Compared with single-organ dysfunction, having 2 or 3 organ dysfunctions was associated with increased risk of long-term mortality (adjusted HR, 1.07; 95% CI, 1.01-1.13; and adjusted HR, 1.18; 95% CI, 1.03-1.14, respectively), while having 4 organ dysfunctions or more was not associated with increased risk. Unexpectedly, the Acute Physiology and Chronic Health Evaluation acute physiology component score had an incremental association with long-term mortality (adjusted HR, 1.11 for every 5-point increase; 95% CI, 1.08-1.13). The adjusted HR for septic shock was 0.89 (95% CI, 0.85-0.92).

**Conclusions and Relevance:**

This study suggests that generic and sepsis-specific risk factors, known during index critical care admission for sepsis, could identify a high-risk sepsis survivor population for biological characterization and designing interventions to reduce long-term mortality.

## Key Points

**Question:**

Which generic and sepsis-specific patient characteristics, known during index critical care admission for sepsis, are independently associated with long-term mortality in sepsis survivors?

**Findings:**

In this cohort study of 94 748 adult sepsis survivors, age, male sex, 1 or more severe comorbidities, prehospitalization dependency, nonsurgical status, acute severity of illness, site of infection, and organ dysfunction were independently associated with long-term mortality.

**Meaning:**

Generic and sepsis-specific risk factors, known during index critical care admission for sepsis, could be used to identify a higher-risk sepsis survivor population for targeted strategies aimed at reducing the excess risk of long-term mortality.

## Teaser

This cohort study investigates the generic and sepsis-specific patient characteristics that are associated with long-term mortality in patients who survive hospitalization for sepsis.

## Introduction

Sepsis is defined as life-threatening organ dysfunction caused by a dysregulated host response to infection.^1^ Sepsis is common, with an estimated population incidence of 148 cases per 100 000 person-years.^2^ Sepsis epidemiology studies from the past 2 decades consistently highlight both increasing incidence and improving acute mortality.^3,4^ The result of this combination is an increasing number of sepsis survivors (defined in this article as adult patients who survived to hospital discharge following a critical care unit admission for sepsis). The ongoing health care needs of adult sepsis survivors are an emerging global health care challenge.^5^

It is also well recognized that sepsis survivors have a greater risk of long-term mortality when compared with patients hospitalized for nonsepsis reasons and the general population.^6,7,8,9^ A systematic review^6^ that included 59 studies and explored the association between sepsis and long-term mortality indicated that, on average, 16% of sepsis survivors die in the first year following their index hospitalization for sepsis. There are major variations in the sepsis case definition and in the risk factors assessed for this long-term mortality between studies. When studying risk factors for long-term mortality in sepsis survivors, the factors associated with short-term mortality may overwhelm and disguise factors associated with long-term mortality—as observed in many sepsis epidemiology studies included in the recent systematic review.^6,10^ The reason for this is that an increase in sepsis severity may worsen cumulative long-term mortality by increasing hospital mortality. This competing risk of hospital mortality on long-term mortality may hide the impact of acute illness characteristics, unless we study a sepsis survivor cohort. Therefore, the following should be considered in the selection of factors to study: (1) potential factors associated with mortality should include both the generic and sepsis-specific factors that resulted in the critical care admission for sepsis^11^; (2) generic factors should relate to the baseline risk of death irrespective of sepsis (eg, severe comorbidity, preadmission dependency status); and (3) sepsis-specific factors should reflect the severity of sepsis. In this context, inferences are not biased when studying a sepsis survivor population (survivorship bias), as the exposure of interest is conditional on surviving to hospital discharge following a critical care admission for sepsis. If long-term mortality risk after sepsis is associated with acute illness characteristics that are known when a sepsis survivor leaves the hospital, those risk factors could be used to identify a target sepsis survivor population for follow-up care, biological characterization, and designing interventions.^5,10^

In this context, we assessed which of the generic (eg, age, sex) and sepsis-specific (eg, site of infection, number of organ dysfunctions) patient characteristics, known during index critical care admission for sepsis, were independently associated with long-term mortality in a sepsis survivor population. To address our research aim, we identified a nationally representative cohort of sepsis survivors from an inception cohort of adults with a critical care admission for sepsis according to the Third International Consensus Definitions for Sepsis and Septic Shock (Sepsis-3)^3^ criteria who survived to hospital discharge in England between April 1, 2009, and March 31, 2014.

## Methods

### Study Database and Approvals

We report an observational cohort study, as per Strengthening the Reporting of Observational Studies in Epidemiology (STROBE) guidelines.^12^ We used data from the Intensive Care National Audit & Research Centre (ICNARC) Case Mix Programme, which is the national clinical audit covering all adult general critical care units in England. For consecutive critical care admissions, trained data collectors collect sociodemographic, comorbidity, and physiological data to precise rules and definitions for the patient’s first 24 hours following admission to critical care. Diagnostic data are determined clinically and coded using a 5-tier, hierarchical ICNARC coding method (eAppendix in the Supplement).^13^ Collected data undergo extensive local and central validation prior to pooling into the Case Mix Programme Database.^14^ Patient-level unique identifiers are also generated for additional data linkage.^13^ Long-term survival status and date of death were ascertained by linking the Case Mix Programme database to the Hospital Episode Statistics database and Office for National Statistics records.^15^ Support for the collection and use of these data has been obtained under Section 251 of the National Health Service Act 2006. Waiver of informed consent for use of this data was obtained from Wales Research Ethics Committee 5 and Confidentiality Advisory Group, which approved this study as part of the Long-term Outcomes After Critical Illness Project.

### Identification of Sepsis Survivor Cohort

As of March 31, 2015, we ascertained the date of death for consecutive admissions to 192 adult, general critical care units in England participating in the ICNARC Case Mix Programme between April 1, 2009, and March 31, 2014. From these, we identified a cohort of Sepsis-3 sepsis survivors, defined as adult patients who survived to hospital discharge following an index critical care admission for sepsis meeting Sepsis-3 criteria. We defined the index critical care admission for sepsis as the hospitalization that included the first critical care unit admission with infection and total Sequential Organ Failure Assessment score of 2 or greater. We defined septic shock as infection, with the cardiovascular component of the Sequential Organ Failure Assessment of 2 or greater and a serum lactate concentration greater than 18 mg/dL (to convert to millimoles per liter, multiply by 0.111), as per Sepsis-3 criteria.^1,16^ We identified presence of infection from the reported primary (mandated) and secondary (optional) reasons for critical care admission.^3^ As baseline organ dysfunction at index critical care admission was unknown for our study cohort, we assumed a baseline organ dysfunction of 0, as recommended by the Sepsis-3 consensus definitions.^1^ We have published this operationalization,^3^ which included sensitivity analyses to confirm results by excluding patients with any preexisting severe comorbidity (eTable 1 in the Supplement).

### Statistical Analysis

The primary outcome was time to death from any cause during follow-up. Generic and sepsis-specific patient characteristics, known during index critical care admission for sepsis, were considered as risk factors, informed by our systematic reviews.^6,10^ Generic patient characteristics included were sepsis discharge year, age, sex, ethnicity, severe comorbidities (defined using the Acute Physiology and Chronic Health Evaluation [APACHE II] method^17^), prehospitalization dependency, surgical status, and acute severity of illness defined using the APACHE II acute physiology component score.^17^ The prehospitalization dependency is a subjective assessment of whether the participant receives no, some, or total assistance to complete daily activities such as bathing, dressing, going to the toilet, moving in and out of a bed or chair, continence, and eating prior to critical care admission. Sepsis-specific patient characteristics included were site of infection, number of organ dysfunctions defined as Sequential Organ Failure Assessment score of 1 or greater per organ, and septic shock status.

First, we generated cumulative time-to-mortality plots for all generic and sepsis-specific patient characteristics and tested equality of mortality functions using a Peto-Peto-Prentice log-rank test. The Peto-Peto-Prentice log-rank test was specifically chosen because it is not affected by differences in censoring patterns across different strata. We then assessed which generic and sepsis-specific patient characteristics were independently associated with long-term mortality in adult sepsis survivors using adjusted hazard ratios (HRs) from proportional hazards (Cox) regression modeling. The categorical variables were sex, race/ethnicity, severe comorbidity, surgical status, preadmission dependency, site of infection, number of organ dysfunctions, and septic shock status. Continuous variables were entry date (index date of discharge from hospital), age (in decades), APACHE II acute physiology component score (in 5-point increments), and index hospital length of stay. A priori, we included an interaction term between the APACHE II acute physiology component score (in 5-point increments) and severe comorbidities. We also estimated time-varying coefficients for age (in decades), APACHE II acute physiology component score (in 5-point increments), and severe comorbidities using an interaction between the covariate and follow-up time that allowed the strength of association between a risk factor and the event of interest to either increase or decrease during the course of follow-up. For all Cox regression models, we tested for evidence against the proportional hazard assumption on the basis of Schoenfeld residuals after fitting a model with Cox regression.^18^ We gave equal weights to different comorbidities for interpretability, accounted for clustering by intensive care units using robust standard errors, did not perform any variable selection approaches, and used the Breslow method for handling ties, which considers each event at a given time as distinct and allows all failed subjects to contribute fully to the risk set (here, *failed subjects* is a technical term referring to how the Breslow method handles ties within the Cox model).

### Sensitivity Analyses

We performed 5 separate sensitivity analyses. First, to account for overlap in variables that contribute toward the APACHE II acute physiology component score and severe comorbidity (eg, creatinine, oxygenation), we performed a sensitivity analysis replicating the Cox regression model in patients without severe comorbidity. Second, to ascertain whether changes observed were influenced by coverage (additional critical care units participating in the Case Mix Programme over time), we repeated all the analyses on a subpopulation of 117 critical care units that contributed data to the Case Mix Programme for the entire study period. Third, to ascertain whether the primary analyses hold true, as in a 5-year cohort study the adjusted HR could change, we report the adjusted HR overlapping time intervals 0 to 1, 0 to 2, 0 to 3, 0 to 4, and 0 to 5 years following hospital discharge. Fourth, to ascertain whether changes observed were influenced by an unobserved random factor between critical care units that modifies the hazard function of an individual or a group or cluster of individuals, we report a mixed-effects Cox model (shared frailty model).^19^ Fifth, to provide direct comparison with recently published cohort studies,^7,8,9^ we estimated the excess long-term mortality in sepsis survivors compared with age-, sex-, and index year–matched expected probabilities of death^20^ in the general population of England between 2009 and 2014^21^ using the relative survival framework with Ederer II estimation.

Reported *P* values are 2-sided, with *P* values less than .05 considered to represent statistically significant results. Continuous data that were normally distributed were summarized as mean and standard deviation. Data that were not normally distributed were summarized as median and interquartile range (IQR). Categorical data were presented as frequency and percentage. Data completeness was extremely good; therefore, a complete case analysis was used, with less than 0.5% of patients excluded from the final model owing to missing data on risk factors. Statistical analyses were completed in June 2017. All analyses were performed using Stata/SE statistical software version 14.0 (StataCorp LP).

## Results

### Study Cohort

**Figure 1.**
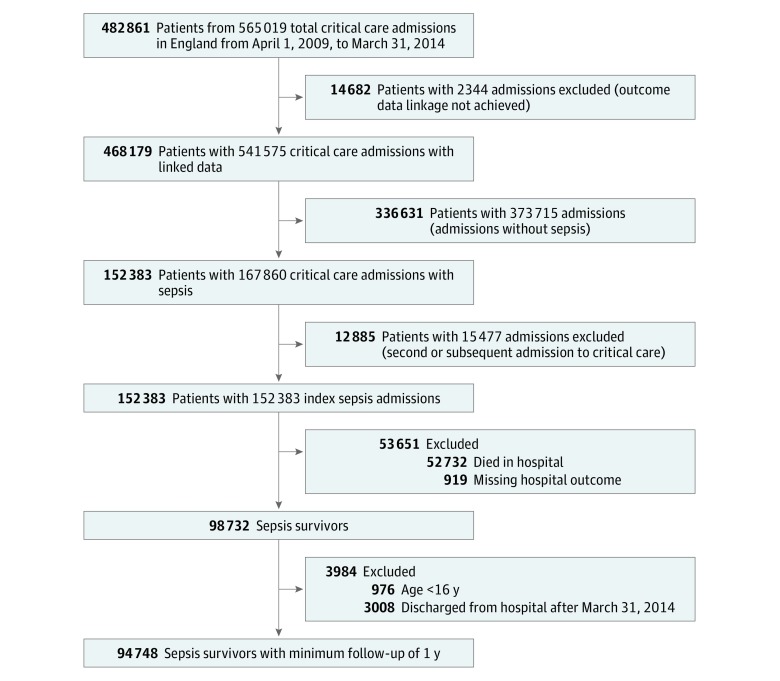
Flow Diagram for Identification of Sepsis Survivor Cohort Sepsis survivors were defined as adult patients who survived to hospital discharge following an index critical care admission with sepsis. We defined the index critical care admission for sepsis as the hospitalization in the study period that included the first critical care unit admission for sepsis as defined by the Third International Consensus Definitions for Sepsis and Septic Shock.^1,3^ Thus, each sepsis survivor was included only once in the study, based on his or her index critical care admission for sepsis. Some patients have 1 or more critical care admissions with and without sepsis during the study period. Patients with nonsepsis admission may be readmitted during the study period for their index (first) critical care admission for sepsis.

Over the 5-year study period (April 1, 2009, to March 31, 2014), there were 152 383 index critical care admissions for sepsis and 98 732 sepsis survivors. After exclusions for age younger than 16 years (976 patients) and those sepsis survivors who were discharged from the hospital after March 31, 2014 (3008 patients), the sepsis survivor study cohort included 94 748 patients (Figure 1; eFigure in the Supplement).

### Cohort Characteristics and Long-term Mortality

**Figure 2.**
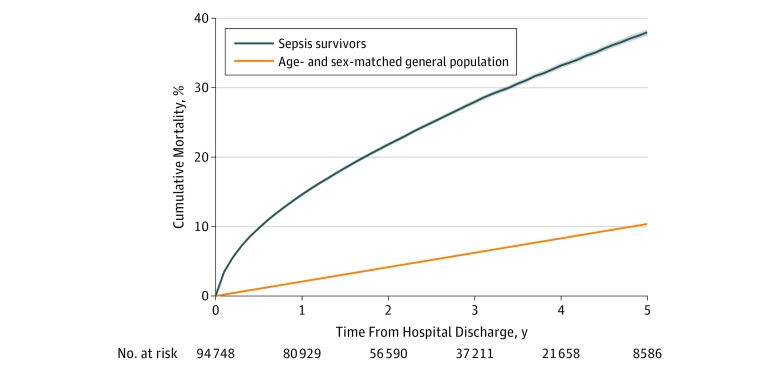
Cumulative All-Cause Long-term Mortality in Adult Sepsis Survivors Compared With Age- and Sex-Matched General Population Cumulative all-cause long-term mortality in the entire cohort of sepsis survivors (as defined by the Third International Consensus Definitions for Sepsis and Septic Shock) compared with age-, sex-, and index sepsis admission year–matched general population expected probabilities of death in England, using relative survival framework (eTable 3 in the Supplement).

**Figure 3.**
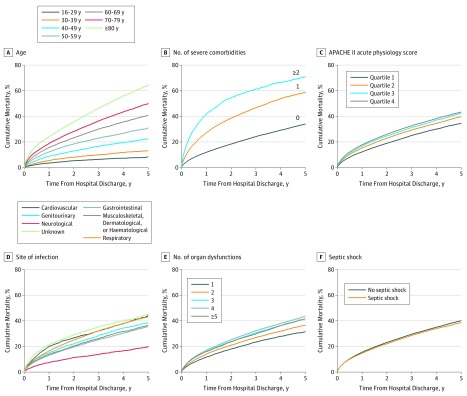
Cumulative All-Cause Long-term Mortality by Risk Factors in Adult Sepsis Survivors A-F, Stratum-level differences in crude mortality of sepsis survivors (as defined by the Third International Consensus Definitions for Sepsis and Septic Shock) over follow-up within generic characteristics (age, ≥1 severe comorbidities, and Acute Physiology and Chronic Health Evaluation II (APACHE II) acute physiology score quartile strata) and sepsis-specific characteristics (site of infection; number of organ dysfunction; and septic shock status) (all *P* < .001 using Peto log-rank test). Number of patients at risk is shown in eTable 4 in the Supplement.

Overall, these sepsis survivors had a mean (SD) age of 61.3 (17.0) years, 43 584 (46.0%) were female, and 86 056 (90.8%) were white. Sepsis survivors more often had nonsurgical admissions (69.3%). The most common site of infection was respiratory (46.3%), the critical care admission day mean (SD) total APACHE II score was 16.7 (5.9) points, and the median (IQR) critical care length of stay was 4 (2-8) days. Further generic and sepsis-specific patient characteristics are reported in the Table. By 1 year after hospital discharge, 15% of sepsis survivors had died, with 6% to 8% dying per year over the next 5 years (Figure 2). Cumulative time-to-mortality plots indicated significant within-strata differences (Figure 3).

**Table.** Characteristics of Sepsis Survivor Cohort^a^

Parameter	No. (%)
Critical care units contributing to data, No.	192
Index sepsis admissions, No.^b^	152 383
In-hospital mortality^c^	52 732 (34.6)
Sepsis survivors with minimum follow-up time of 1 y following hospital discharge^b^	94 748 (100)
Sepsis survivors whose discharge disposition was normal residence	83 378 (88.0)
Age, mean (SD), y	61.3 (17.0)
Female	43 584 (46.0)
Race/ethnicity	
White	86 056 (90.8)
Asian	3378 (3.6)
Black	2020 (2.1)
Other^d^	3924 (3.5)
Severe comorbidity^e^	
0	80 461 (84.9)
1	11 097 (11.7)
≥2	3190 (3.4)
Preadmission dependency	
No dependency	70 737 (75.0)
Some dependency	22 427 (23.8)
Totally dependent	1187 (1.3)
Admission type	
Nonsurgical (medical)	65 691 (69.3)
Elective surgical	5526 (5.8)
Emergency surgical	23 522 (24.8)
Total Acute Physiology and Chronic Health Evaluation II score, mean (SD)	16.7 (5.9)
Acute Physiology and Chronic Health Evaluation II acute physiology component score, mean (SD)	12.4 (5.2)
Site of infection	
Respiratory	43 858 (46.3)
Gastrointestinal	28 630 (30.2)
Cardiovascular	1612 (1.7)
Genitourinary	6747 (7.1)
Musculoskeletal, dermatological, or hematological	5075 (5.4)
Neurological	3206 (3.4)
Unknown	5620 (5.9)
Organ dysfunction, No.^f^	
1	9727 (10.3)
2	26 878 (28.4)
3	31 119 (32.8)
4	21 075 (22.2)
≥5	5949 (6.3)
Septic shock	13 276 (14.0)
Length of stay, median (IQR), d	
Critical care	4 (2-8)
Hospital	21 (11-40)
Follow-up, median (IQR) [maximum], d	893 (499-1409) [2172]
Mortality during follow-up, No./No. (%)^g^	
0-1 y	13 819/94 748 (14.6)
1-2 y	5163/62 358 (8.3)
2-3 y	3057/41 000 (7.5)
3-4 y	1628/23 893 (6.8)
4-5 y	662/9722 (6.8)

Abbreviation: IQR, interquartile range.

^a^ As defined by the Third International Consensus Definitions for Sepsis and Septic Shock criteria.

^b^ Figure 1 shows exclusions (976 patients aged <16 years; 3008 patients with hospital discharge after March 31, 2014).

^c^ eFigure 2 in the Supplement shows the proportion of in-hospital deaths.

^d^ Other includes mixed race/ethnicity or not stated.

^e^ Comorbidities using Acute Physiology and Chronic Health Evaluation II method.^17^

^f^ As defined by Sequential Organ Failure Assessment score.

^g^ The denominator indicates patients who survived the previous interval, then either died or survived but had the full follow-up for that interval, resulting in 18 571 patients between 1 to 2 years; 16 195 between 2 to 3 years; 14 050 between 3 to 4 years; and 12 543 between 4 to 5 years excluded from the denominator.

### Factors Independently Associated With Long-term Mortality

**Figure 4.**
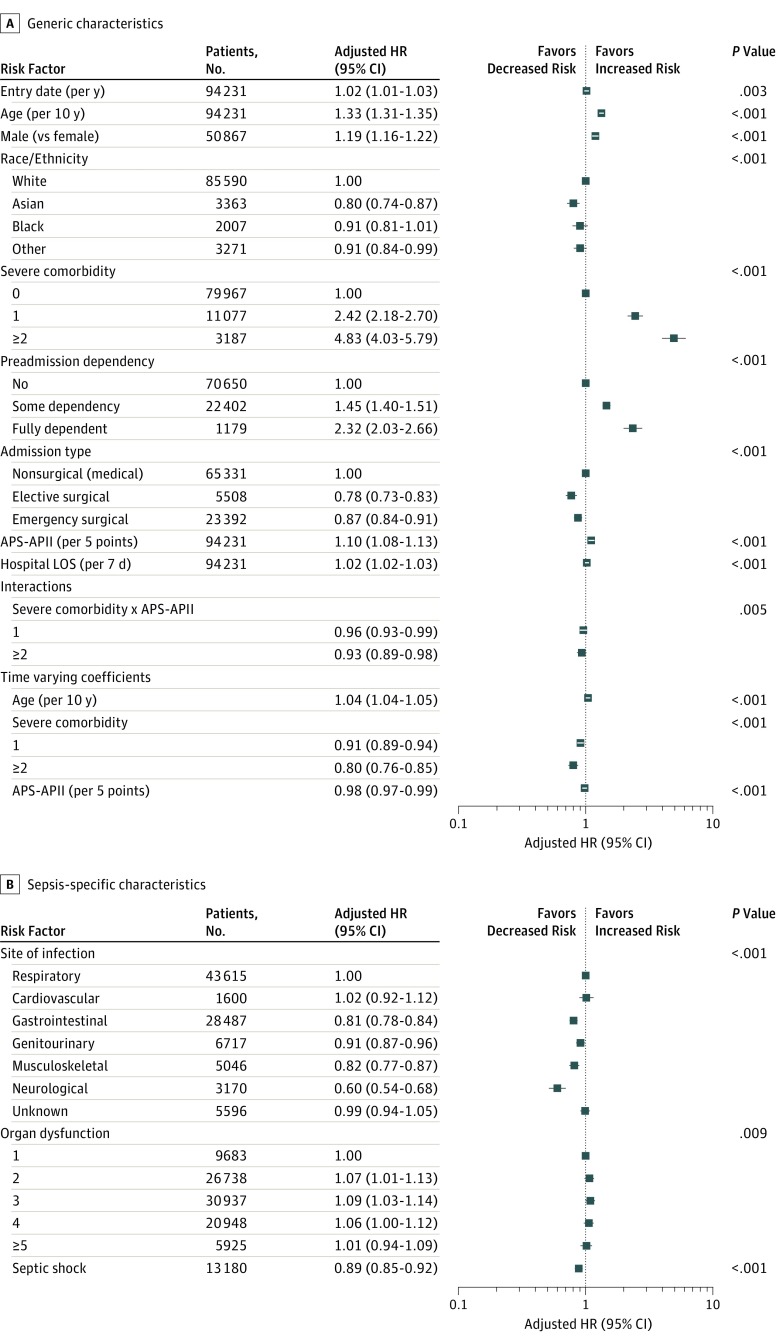
Adjusted Hazard Ratios (HRs) for Each Risk Factor on Long-term Mortality by Survival Time in Adult Sepsis Survivors Adjusted HRs generated using the primary Cox regression model (eTable 2 in the Supplement) are shown for generic risk factors, time-varying coefficients (for age in decades, acute physiology component of Acute Physiology and Chronic Health Evaluation II [APACHE II] score per 5-point increments, and severe comorbidity) and interactions (A) and sepsis-specific risk factors (B). The *P* values represent test for homogeneity within groups, estimated using a postestimation command to look for significant differences after the Cox regression model. See the Methods section for the base category for each risk factor. Sepsis was defined according to the criteria of the Third International Consensus Definitions for Sepsis and Septic Shock. APII-APS indicates acute physiology component of APACHE II; and LOS, length of stay.

Among the generic patient characteristics assessed, increasing age, being male, having 1 or more severe comorbidities, having some or total dependency prior to index critical care admission, and longer index hospital length of stay increased the risk of long-term mortality in sepsis survivors, consistent with published literature. Compared with sepsis survivors who survived their index critical care admission for sepsis between April 2009 and March 2010, sepsis survivors in later years had higher risk of long-term mortality (adjusted HR, 1.02; 95% CI, 1.01-1.03, equating to 2% increase in hazard per year; *P* < .001). Increasing score on the APACHE II acute physiology component was significantly associated with increased risk of long-term mortality (adjusted HR, 1.11 for every 5 points; 95% CI, 1.08-1.13; *P* < .001). Patients with surgical status had a lower risk of long-term mortality than those with nonsurgical (medical) status (Figure 4; eTable 2 in the Supplement).

Among the sepsis-specific characteristics assessed, when compared with respiratory site of infection, gastrointestinal, genitourinary, musculoskeletal, and neurological sites of infection had adjusted HRs for long-term mortality less than 1. Compared with single organ dysfunction, having either 2 or 3 organ dysfunctions was associated with greater mortality (adjusted HR, 1.07; 95% CI, 1.01-1.13; and adjusted HR, 1.18; 95% CI, 1.03-1.14, respectively), while having 4 or more organ dysfunctions was not. Septic shock at index admission had an adjusted HR of 0.89 (95% CI, 0.85-0.92; *P* < .001) for long-term mortality in sepsis survivors (Figure 4; eTable 2 in the Supplement).

### Factors That Modify the Association of Acute Severity of Illness With Long-term Mortality

The association of APACHE II acute physiology component score on longer-term mortality decreased by 4% per year for sepsis survivors with 1 comorbidity and 7% per year for those with 2 or more severe comorbidities. We observed a 4% increase in time-varying hazard per year for age. The time-varying reduction in the hazard for the APACHE II acute physiology component score was less than 2% per year, compared with the 9% decrease in hazard per year for 1 severe comorbidity and 20% decrease in hazard per year for 2 or more severe comorbidities, implying patients with more comorbidities died earlier during follow-up (Figure 4; eTable 2 in the Supplement).

### Sensitivity Analyses

Results from sensitivity analyses were consistent with the primary analyses for the generic and sepsis-specific risk factors assessed. For example, all sensitivity analyses had similar adjusted HRs for increments in the APACHE II acute physiology component score (1) in sepsis survivors without severe comorbidities (n = 80 139; adjusted HR, 1.11 for every 5 points; 95% CI, 1.08-1.13), (2) from 117 critical care units that contributed data over the entire study period (n = 68 703; adjusted HR, 1.10 for every 5 points; 95% CI, 1.07-1.13), (3) for overlapping survival-time intervals, and (4) for random variation with shared frailty model (adjusted HR, 1.08 for every 5 points; 95% CI, 1.07-1.10) (eTable 2 in the Supplement). The relative survival analysis was comparable to previous reports, with survival decreasing from 87.3% by 1 year to 69.2% by 5 years (eTable 3 in the Supplement).

## Discussion

In this cohort of adult sepsis survivors, as defined by Sepsis-3 criteria, both generic and sepsis-specific patient characteristics known during the index critical care admission for sepsis were independently associated with long-term mortality. Increasing age, male sex, 1 or more severe comorbidities, prehospitalization dependency, and nonsurgical (medical) status increased the risk of long-term mortality. The risk of long-term mortality differed by site of infection at index critical care admission for sepsis. Novel findings were the incremental increase in risk of long-term death in sepsis survivors associated with increased APACHE II acute physiology component score (adjusted HR, 1.11; 95% CI, 1.08-1.13 for every 5 points), lower risk associated with septic shock (adjusted HR, 0.89; 95% CI, 0.85-0.92), and the variation in additional hazard for long-term death associated with number of organ dysfunctions. The greater number of in-hospital deaths in patients with septic shock and in patients with greater organ dysfunction is the most likely explanation for these results,^3,11^ an important finding that is often overlooked when estimated using cumulative mortality.

One of the key reasons why we studied a sepsis survivor population was to unmask the important risk factors associated with long-term mortality, which are often disguised by the risk factors associated with short-term mortality, such as acute illness severity. The incremental increase in hazard seen with increasing APACHE II acute physiology component score persisted in the sensitivity analysis, excluding patients with chronic severe comorbidity. This finding, when considered in the context of clinical^11^ and biological^22^ differences within sepsis cohorts, reinforces the value of how routinely available clinical information on index critical care admission for sepsis could inform follow-up care of sepsis survivors. This reasoning is strengthened when noting the differences in the impact of site of infection on long-term mortality we observed, as site of infection potentially influences immune responses.^23^ A related argument in this context is that even in 2 patients with similar predicted risk but with differences in relative contributions of generic and sepsis-specific risk components, differences would exist in treatability of long-term sequalae. Thus, when these factors independently associated with long-term mortality from our results are considered with those from the literature,^5,6,8,10,24,25^ it indicates that sepsis survivors both retain risk from index critical care admission for sepsis but also accumulate additional risk following hospital discharge from their worsening preexisting comorbidities^26^ and/or new comorbidities (eg, worsening cognition,^24^ cardiovascular morbidity^27^), both of which are associated with increased risk of long-term mortality. Thus, our study makes a case for understanding how management following discharge could alter sepsis survivors’ risk of long-term adverse outcomes.^26^

### Strengths

We used the Case Mix Programme database, which is a national database for critical care units in England and has been independently assessed to be of high quality.^14^ We reduced selection bias by studying consecutive critical care admissions for sepsis from 192 critical care units representing the whole critical care population in a country over a 6-year period and commencing patient follow-up from hospital discharge with 96% data linkage for long-term mortality. Long-term mortality from a single health care system is often challenged on external validity owing to differences in clinical care.^28^ We have maximized the external validity of our study by using a valid Sepsis-3 sepsis case definition in a nationally representative adult sepsis survivor cohort older than 16 years. These are important strengths, as, of the 2 recent long-term sepsis outcome studies, 1 focused on older adults (≥65 years) with insurance coverage admitted between 2002 and 2010,^8^ and the second reported sepsis cases between 2000 and 2002 from a national health insurance database.^7^

### Limitations

This study has limitations. As seen in all follow-up studies, our cohort had different durations of follow-up. We used proportional hazards modeling to account for differences in follow-up duration and adjusted for common confounders^6,10^ in the association between sepsis and long-term mortality based on systematic review of literature.^6^ Our study cohort reports the epidemiology of adult sepsis survivors, who all had critical care admissions with sepsis identified in the first 24 hours of admission. Thus, we will underestimate patients with nonsepsis critical care admissions who develop sepsis later in their critical care stay. However, as England has among the lowest per capita critical care bed provision in Europe (3.5-7.4 per 100 000 population),^29^ probability of underestimation is low, as organ dysfunction will often be present on admission day. We were not able to study the biological mechanisms that could explain our findings. Information bias due to misclassification of sepsis exposure or long-term mortality is a risk that we reduced by deriving sepsis cases using raw physiology and long-term mortality from a high-quality national data source. As our goal was to identify risk factors, we have only reported age- and sex- matched general population controls for comparison with those studies that quantify excess long-term risk of death from sepsis.^7,8,9^ A marker of physiological disturbance closer to discharge would be potentially useful, with the counterpoints that the median critical care length of stay was 4 days and if there was significant physiological disturbance, patients would not be discharged from the hospital. We do not present a risk score to implement at bedside, as this was not our research question. Although we did not estimate a formal sample size calculation, we do report the long-term mortality from the largest adult sepsis survivor cohort to date.

Our observations that acute illness severity and site of infection at index admission are associated with increased risk of long-term mortality in sepsis survivors has biological plausibility and informs future research. Acute illness severity and illness trajectory seen with critical care admissions for sepsis are associated with health before the illness causing hospitalization, immune responses in sepsis,^22,30,31^ and early appropriate clinical management.^32,33,34^ Immune abnormalities seen in patients with sepsis^31^ appear to persist in sepsis survivors.^35,36^ New or relapsed infection is one of the common reasons for rehospitalization in sepsis survivors, which is potentially associated with site of infection at index sepsis admission.^10,37^ Thus, observational studies identifying biological characteristics and value of parsimonious clinical prediction tools using generic and sepsis-specific risk factors to predict risk of long-term adverse health in sepsis survivors would be valuable. The future interventional trials may include clinical practice interventions such as extended follow-up care either by primary care physicians^5^ or with targeted follow-up clinics^38^ or immunological interventions informed by acute sepsis illness biology.^39,31,40^ Our study adds further impetus to the recently adopted World Health Organization resolution calling for further work informing “policy decisions related to preventive, diagnostic and treatment activities and access to relevant health care for sepsis survivors.”^41^

## Conclusions

Generic and sepsis-specific risk factors, known during index critical care admission for sepsis, are important risk factors associated with long-term mortality seen in a sepsis survivor cohort. Our research provides validity to target sepsis survivor populations based on index admission characteristics, for biological characterization and designing interventions to reduce long-term mortality.

## Notes

Supplement. **eAppendix.** Further Description of Methods **eFigure.** Stacked Graph of In-Hospital Mortality, Post-Hospital Mortality at One Year and Survival Proportions **eTable 1.** Operationalization of Sepsis-3 Definitions **eTable 2.** Primary Model, and Four Sensitivity Analyses **eTable 3.** Relative Survival Over Time for Sepsis Survivors **eTable 4.** Number of Patients at Risk **eReferences**
