# Supplementary material for: Risk Factors at Index Hospitalization Associated With Longer-term Mortality in Adult Sepsis Survivors
Source: JAMA Netw Open. 2019 May 31;2(5):e194900. doi: 10.1001/jamanetworkopen.2019.4900 (PMC6547123; doi:10.1001/jamanetworkopen.2019.4900)
Supplement: Supplement. — eAppendix. Further Description of Methods eFigure. Stacked Graph of In-Hospital Mortality, Post-Hospital Mortality at One Year and Survival Proportions eTable 1. Operationalization of Sepsis-3 Definitions eTable 2. Primary Model, and Four Sensitivity Analyses eTable 3. Relative Survival Over Time for Sepsis Survivors eTable 4. Number of Patients at Risk eReferences [file jamanetwopen-2-e194900-s001.pdf]

Supplementary Online Content

Shankar-Hari M, Harrison DA, Ferrando-Vivas P, Rubenfeld GD, Rowan K. Risk factors at index hospitalization associated with longer-term mortality in adult sepsis survivors. *JAMA Netw Open*. 2019;2(5):e194900. doi:10.1001/jamanetworkopen.2019.4900

eAppendix. Further Description of Methods
eFigure. Stacked Graph of In-Hospital Mortality, Post-Hospital Mortality at One Year and Survival Proportions
eTable 1. Operationalization of Sepsis-3 Definitions
eTable 2. Primary Model, and Four Sensitivity Analyses
eTable 3. Relative Survival Over Time for Sepsis Survivors
eTable 4. Number of Patients at Risk
eReferences

This supplementary material has been provided by the authors to give readers additional information about their work.

© 2019 Shankar-Hari M et al. *JAMA Network Open.*

eAppendix. Further description of methods

Systematic review to inform analysis:

We recently published a systematic review to inform our analytic approach[1]. We assessed how individual studies defined the exposure and addressed confounding (stratification, regression models or controls) for the sepsis – mortality at one-year post-hospital discharge relationship. The mean (95% confidence interval) one-year post-hospital discharge mortality was 16.1 % (14.1, 18.1 %) with significant heterogeneity ($I^2 = 98.9$ %; $p < 0.001$; random effects meta-analysis of 43 studies). Since our systematic review, two further papers have reported longer-term mortality[2,3], consistent with the findings from our systematic review.

Description of the ICNARC coding method [4]

Diagnostic data are determined clinically and coded using a five-tier, hierarchical ICNARC Coding Method: type, system, site, process and condition.

- Type: is it a surgical code (reason for surgery) or non-surgical code?
- System: Which body system is involved? This refers to body systems such as respiratory, cardiovascular, gastrointestinal and others.
- Site: Which anatomical site is involved? For example, for the respiratory tract, this will include descriptions such as trachea, bronchi, lung, pleura and others
- Process: Which physiological or pathological process is involved? This refers to abnormal processes such as infection, collapse, perforation, rupture and others.
- Condition: What is the name of the condition? This refers to the clinical condition. For example, a patient with pneumonia will have the following hierarchical codes:
  - Type = non-surgical
  - System = respiratory
  - Site = lung
  - Process = infection
  - Condition = Pneumonia

Sepsis case definition:

Infection was identified using the primary or secondary reason for critical care admission. Description of how site of infection is coded in the dataset is explained above using the ICNARC coding method. We have published this operationalisation, which also included sensitivity analyses to confirm results by excluding patients with a pre-existing comorbidity (eTable-1).[5]

© 2019 Shankar-Hari M et al. *JAMA Network Open.*

eFigure: Stacked graph of in-hospital mortality, post-hospital mortality at one year and survival proportions

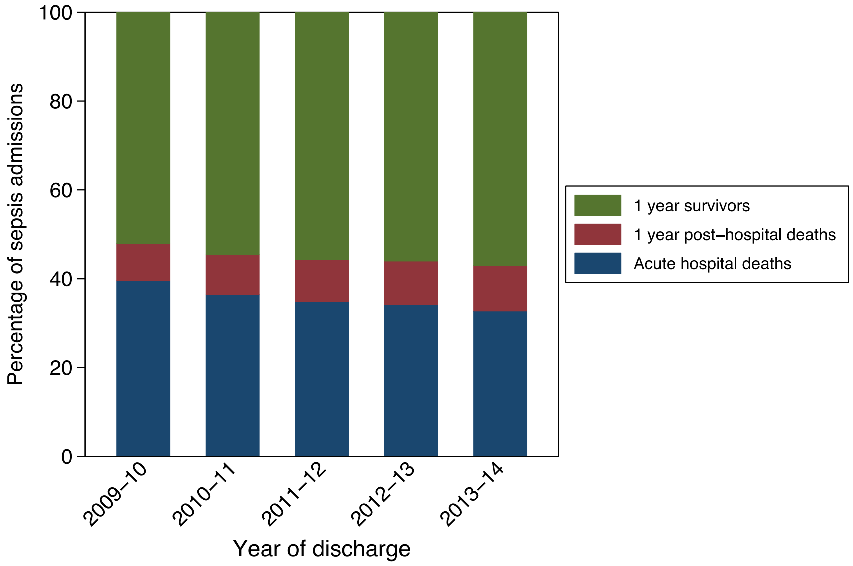

We have previously reported an increase in Sepsis-3 sepsis incidence and significantly improving trends in hospital mortality in England, from 33.6% to 30.5% (adjusted odds ratio = 0.96(0.95–0.97); p<0.001)[5]. Due to greater acute severity of illness, more sepsis-3 septic shock patients die during their index hospitalisation for a sepsis-related critical illness [5].

In the current study, we report the longer-term mortality of the Sepsis-3 sepsis patients, who survived this index sepsis-related ICU admission, in the context of significantly improving acute mortality.

eTable 1: Operationalization of Sepsis-3 definitions

Criteria	Sepsis-3
Infection	Primary or secondary reason for ICU admission
Organ dysfunction	SOFA score of 2 or more in any one organ system or SOFA score of 1 in two or more organ systems Modified SOFA score was derived as described in the legend
Sepsis	Sepsis = Infection AND >=2 SOFA points
Septic shock	Infection AND cardiovascular SOFA>=2 AND serum lactate concentration>2mmol/L

We have published this operationalisation, which also included sensitivity analyses to confirm results by excluding patients with a pre-existing comorbidity.[5]

Adapted from Shankar-Hari M, Harrison DA, Rubenfeld GD, Rowan K. Epidemiology of sepsis and septic shock in critical care units: comparison between sepsis-2 and sepsis-3 populations using a national critical care database. *BJA: British Journal of Anaesthesia* 2017; **119**(4): 626-36.

© 2019 Shankar-Hari M et al. *JAMA Network Open.*

eTable 2: Primary model, and four sensitivity analyses

Covariate	Primary model	Time-horizons analysis					Units contributing throughout*	No comorbidities	Shared Frailty model
		0 - 5 years	0 - 4 years	0 - 3 years	0 - 2 years	0 - 1 year			
Cohort entry time (per year)	1.02 (1.01, 1.03)	1.02 (1.01, 1.03)	1.02 (1.00, 1.03)	1.01 (1.00, 1.02)	1.00 (0.99, 1.02)	1.00 (0.99, 1.02)	1.02 (1.00, 1.03)	1.01 (1.00, 1.02)	1.01 (1.00, 1.02)
Age (per 10 year increase)	1.33 (1.31, 1.35)	1.33 (1.31, 1.35)	1.33 (1.31, 1.36)	1.34 (1.32, 1.37)	1.36 (1.34, 1.39)	1.39 (1.36, 1.43)	1.34 (1.32, 1.36)	*1.43 (1.40, 1.45)*	1.41 (1.40, 1.43)
Male (vs female)	1.19 (1.16, 1.22)	1.19 (1.16, 1.22)	1.19 (1.16, 1.22)	1.18 (1.15, 1.22)	1.18 (1.14, 1.22)	1.17 (1.13, 1.22)	1.20 (1.16, 1.24)	1.21 (1.18, 1.25)	1.19 (1.16, 1.22)
Ethnicity (vs white)									
Asian	0.80 (0.74, 0.87)	0.80 (0.74, 0.87)	0.81 (0.74, 0.88)	0.82 (0.75, 0.89)	0.83 (0.75, 0.91)	0.85 (0.76, 0.96)	0.78 (0.71, 0.85)	0.80 (0.73, 0.88)	0.76 ().70, 0.82)
Black	0.90 (0.81, 1.01)	0.90 (0.81, 1.00)	0.90 (0.81, 1.01)	0.93 (0.83, 1.04)	0.93 (0.82, 1.05)	0.94 (0.82, 1.08)	0.87 (0.76, 0.99)	0.81 (0.71, 0.92)	0.84 (0.76, 0.92)
Other	0.91 (0.83, 0.99)	0.91 (0.84, 0.99)	0.92 (0.84, 1.00)	0.94 (0.86, 1.02)	0.95 (0.86, 1.04)	0.92 (0.83, 1.03)	0.92 (0.82, 1.02)	0.91 (0.83, 1.01)	0.88 (0.81, 0.94)
Severe comorbidities (vs none)									
1	2.42 (2.18, 2.70)	2.41 (2.17, 2.69)	2.40 (2.15, 2.69)	2.46 (2.18, 2.78)	2.44 (2.16, 2.77)	2.44 (2.10, 2.84)	2.40 (2.12, 2.71)	n/a	2.16 (1.98, 2.43)
2 or more	4.83 (4.03, 5.79)	4.87 (4.07, 5.84)	5.00 (4.17, 6.00)	5.18 (4.29, 6.25)	5.21 (4.26, 6.38)	5.46 (4.32, 6.90)	4.79 (3.90, 5.80)	n/a	3.81 (3.33, 4.35)
Dependency (vs none)									
Some (minor or major)	1.45 (1.40, 1.51)	1.45 (1.40, 1.51)	1.44 (1.38, 1.50)	1.42 (1.37, 1.48)	1.41 (1.35, 1.47)	1.41 (1.34, 1.47)	1.46 (1.39, 1.53)	1.65 (1.59, 1.72)	1.49 (1.45, 1.53)
Total	2.33 (2.03, 2.66)	2.33 (2.04, 2.66)	2.32 (2.03, 2.65)	2.29 (2.00, 2.63)	2.25 (1.94, 2.62)	2.30 (1.95, 2.72)	2.28 (1.93, 2.70)	2.78 (2.36, 3.26)	2.37 (2.15, 2.60)
Surgical status (vs non-surgical)									
Elective/scheduled	0.78 (0.73, 0.83)	0.77 (0.72, 0.83)	0.76 (0.72, 0.83)	0.75 (0.70, 0.81)	0.75 (0.68, 0.81)	0.74 (0.67, 0.82)	0.77 (0.71, 0.83)	0.77 (0.71, 0.83)	0.78 (0.73, 0.83)
Emergency/urgent	0.87 (0.84, 0.91)	0.87 (0.84, 0.91)	0.87 (0.83, 0.90)	0.87 (0.83, 0.91)	0.86 (0.82, 0.91)	0.88 (0.83, 0.94)	0.88 (0.84, 0.92)	0.87 (0.84, 0.91)	0.89 (0.85, 0.92)
Site of infection (vs respiratory)									
Cardiovascular	1.02 (0.92, 1.12)	1.01 (0.92, 1.12)	1.01 (0.92, 1.12)	1.02 (0.92, 1.13)	1.08 (0.97, 1.20)	1.21 (1.06, 1.37)	1.05 (0.94, 1.20)	1.01 (0.89, 1.13)	1.02 (0.94, 1.12)
Gastrointestinal	0.81 (0.78, 0.84)	0.81 (0.78, 0.84)	0.82 (0.79, 0.85)	0.82 (0.79, 0.86)	0.84 (0.81, 0.89)	0.87 (0.82, 0.92)	0.82 (0.78, 0.86)	0.74 (0.71, 0.78)	0.82 (0.79, 0.85)
Genitourinary	0.91 (0.87, 0.96)	0.91 (0.87, 0.96)	0.93 (0.88, 0.97)	0.92 (0.88, 0.96)	0.93 (0.88, 0.98)	0.96 (0.90, 1.03)	0.90 (0.85, 0.95)	0.85 (0.81, 0.90)	0.92 (0.88, 0.97)
Musculoskeletal/dermatological/ Haematological	0.82 (0.77, 0.87)	0.82 (0.77, 0.87)	0.82 (0.77, 0.88)	0.82 (0.77, 0.88)	0.84 (0.78, 0.91)	0.88 (0.80, 0.97)	0.82 (0.76, 0.88)	0.77 (0.71, 0.82)	0.83 (0.78, 0.88)
Neurological	0.60 (0.53, 0.68)	0.60 (0.53, 0.68)	0.60 (0.53, 0.68)	0.60 (0.53, 0.68)	0.64 (0.56, 0.73)	0.66 (0.57, 0.77)	0.59 (0.51, 0.68)	0.56 (0.50, 0.64)	0.61 (0.56, 0.67)
Unknown	0.99 (0.94, 1.04)	0.99 (0.94, 1.05)	1.00 (0.95, 1.06)	1.02 (0.97, 1.09)	1.01 (1.00, 1.13)	1.14 (1.06, 1.23)	0.96 (0.90, 1.02)	0.89 (0.84, 0.96)	0.99 (0.94, 1.04)
Septic shock (vs none)	0.89 (0.85, 0.92)	0.88 (0.85, 0.92)	0.89 (0.86, 0.93)	0.89 (0.86, 0.93)	0.88 (0.84, 0.92)	0.88 (0.84, 0.93)	0.90 (0.86, 0.94)	0.89 (0.86, 0.93)	0.88 (0.85, 0.92)

© 2019 Shankar-Hari M et al. *JAMA Network Open.*

Organ dysfunction (vs One)									
2	1.07 (1.01, 1.13)	1.07 (1.01, 1.13)	1.06 (1.01, 1.12)	1.07 (1.01, 1.29)	1.07 (1.00, 1.14)	1.08 (1.01, 1.16)	1.06 (1.00, 1.14)	1.09 (1.03, 1.16)	1.07 (1.01, 1.12)
3	1.08 (1.03, 1.14)	1.08 (1.03, 1.14)	1.08 (1.02, 1.14)	1.08 (1.02, 1.14)	1.08 (1.01, 1.14)	1.08 (1.02, 1.16)	1.12 (1.05, 1.19)	1.10 (1.03, 1.17)	1.08 (1.07, 1.10)
4	1.06 (1.00, 1.12)	1.06 (1.00, 1.12)	1.05 (0.99, 1.11)	1.05 (0.99, 1.12)	1.05 (0.98, 1.12)	1.05 (0.97, 1.13)	1.10 (1.02, 1.16)	1.05 (0.98, 1.13)	1.05 (1.00, 1.11)
5 or more	1.01 (0.95, 1.09)	1.01 (0.94, 1.09)	1.00 (0.93, 1.08)	0.99 (0.91, 1.07)	0.98 (0.90, 1.06)	0.98 (0.89, 1.09)	1.04 (0.96, 1.13)	0.99 (0.91, 1.07)	1.01 (0.94, 1.08)
LOS in acute hospital (per week)	1.02 (1.02, 1.03)	1.02 (1.02, 1.03)	1.02 (1.02, 1.03)	1.02 (1.02, 1.03)	1.03 (1.02, 1.03)	1.03 (1.02, 1.03)	1.03 (1.02, 1.03)	1.03 (1.02, 1.03)	1.02 (1.02, 1.04)
APACHE II APS per 5-points	1.11 (1.08, 1.13)	1.11 (1.08, 1.13)	1.11 (1.09, 1.14)	1.12 (1.10, 1.15)	1.13 (1.10, 1.16)	1.15 (1.12, 1.19)	1.10 (1.07, 1.13)	1.11 (1.08-1.13)	1.08 (1.07, 1.10)
Interaction between severe conditions in PMH and APII-APS (vs no comorbidity)									
1	0.96 (0.93, 0.99)	0.96 (0.93, 1.00)	0.96 (0.93, 1.00)	0.96 (0.93, 1.00)	0.96 (0.92, 1.00)	0.94 (0.90, 0.99)	0.97 (0.92, 1.00)	n/a	0.96 (0.94, 0.99)
2 or more	0.93 (0.89, 0.98)	0.93 (0.89, 0.98)	0.93 (0.88, 0.98)	0.92 (0.87, 0.97)	0.91 (0.86, 0.97)	0.88 (0.82, 0.94)	0.93 (0.88 – 0.99)	n/a	0.94 (0.90, 0.98)
Time-varying Coefficients<br>Age in 10yr increments<br>Comorbidity (vs no)<br>1<br>2 or more<br>APII-APS per 5 points	1.04 (1.04, 1.05)<br>0.91 (0.89, 0.94)<br>0.80 (0.76, 0.85)<br>0.98 (0.97, 0.99)	1.04 (1.04, 1.05)<br>0.91 (0.89, 0.94)<br>0.79 (0.75, 0.84)<br>0.98 (0.97, 0.99)	1.04 (1.03, 1.05)<br>0.92 (0.89, 0.95)<br>0.78 (0.74, 0.83)<br>0.98 (0.97, 0.99)	1.03 (1.02, 1.04)<br>0.90 (0.86, 0.93)<br>0.77 (0.71, 0.82)<br>0.96 (0.95, 0.98)	1.01 (0.99, 1.03)<br>0.92 (0.87, 0.98)<br>0.81 (0.74, 0.90)<br>0.96 (0.94, 0.98)	0.94 (0.90, 0.99)<br>1.10 (0.96, 1.25)<br>0.97 (0.79, 1.20)<br>0.92 (0.87, 0.97)	1.04 (1.03, 1.05)<br>0.92 (0.89, 0.95)<br>0.81 (0.76, 0.87)<br>0.98 (0.97, 1.00)	1.03 (1.02, 1.04)<br>n/a<br>n/a<br>0.98 (0.97, 0.99)	n/a<br>n/a<br>n/a<br>n/a

© 2019 Shankar-Hari M et al. *JAMA Network Open.*

eTable 2: Primary model, model with time varying coefficient and sensitivity analyses (a) Time horizons analysis; (b) with 117 intensive care units contributing data throughout the cohort over the entire study period (c) patients without comorbidities and (d) shared frailty model. * 68,703 admissions to 117 of the 192 critical care units; 80,139 sepsis survivors without severe comorbidities at index admission; Italicised text indicates hazard ratios with 95% confidence intervals that exclude the point estimate from the primary model

In all models, the baseline category for sex was female; baseline category for past medical history status was no past medical history; baseline categories for acute admission type was non-surgical (medical) ; baseline category for site of infection was respiratory; baseline category for dependency was no dependency baseline category for organ dysfunction was one organ dysfunction; baseline category for sepsis shock was no septic shock. Abbreviations - tmy = time in years; agedec – age in 10-year increments; pmhp = past medical history present; ethng = Ethnicity; ap2aps = APACHE II acute physiology score; site of infection CA= cardiovascular; GA= gastrointestinal; GE = genitourinary; MD = musculoskeletal and haematological; NE = neurological and UK = unknown; tvc = time varying coefficients

eTable 3: Relative survival over time for sepsis survivors

Survival time	Overall (N=94,748) % (95% CI)
1 Years	87.3 (87.0 – 87.5)
2 Years	81.7 (81.4 – 81.9)
3 Years	77.0 (76.7 – 77.3)
4 years	73.1 (72.7 – 73.5)
5 years	69.7 (69.2 – 70.1)

The relative survival frameworks using Ederer II estimation calculates the expected survival rates amongst sepsis survivors considering the matched general population individuals to be at risk until the corresponding sepsis patient dies or is censored [6]. Sepsis survivors had a significantly higher longer-term mortality compared with age, sex and admission year-matched general population (Figure-1), with reducing survival from 87.3% by one-year to 69.2% by five-years

© 2019 Shankar-Hari M et al. *JAMA Network Open.*

eTable 4: Number of patients at risk

Categories	time in years	At risk	Follow-up	fail	failure	Lower bound	Upper bound
Overall	0	94748	94748	13819	14.6%	14.4%	14.8%
	1	80929	62537	5163	8.3%	8.0%	8.5%
	2	56590	41000	3057	7.5%	7.2%	7.7%
	3	37211	23893	1628	6.8%	6.5%	7.1%
	4	21658	9722	662	6.8%	6.3%	7.3%
	5	8586	0	0	.	.	.
**Age in years**							
16-29	0	5683	5683	196	3.4%	3.0%	4.0%
	1	5487	4340	77	1.8%	1.4%	2.2%
	2	4195	3158	32	1.0%	0.7%	1.4%
	3	3076	2083	15	0.7%	0.4%	1.2%
	4	2020	926	9	1.0%	0.5%	1.8%
	5	871	0	0	.	.	.
30-39	0	6151	6151	328	5.3%	4.8%	5.9%
	1	5823	4613	125	2.7%	2.3%	3.2%
	2	4426	3284	65	2.0%	1.6%	2.5%
	3	3166	2156	37	1.7%	1.2%	2.4%
	4	2054	910	16	1.8%	1.1%	2.8%
	5	848	0	0	.	.	.
40-49	0	10731	10731	928	8.6%	8.1%	9.2%
	1	9803	7641	345	4.5%	4.1%	5.0%
	2	7224	5243	202	3.9%	3.4%	4.4%
	3	4948	3237	108	3.3%	2.8%	4.0%
	4	3040	1332	45	3.4%	2.5%	4.5%
	5	1217	0	0	.	.	.
50-59	0	14830	14830	1782	12.0%	11.5%	12.5%
	1	13048	10101	706	7.0%	6.5%	7.5%
	2	9274	6732	356	5.3%	4.8%	5.8%
	3	6256	4020	184	4.6%	4.0%	5.3%
	4	3724	1673	90	5.4%	4.4%	6.6%
	5	1502	0	0	.	.	.
60-69	0	22380	22380	3443	15.4%	14.9%	15.9%
	1	18937	14538	1268	8.7%	8.3%	9.2%
	2	13067	9405	800	8.5%	8.0%	9.1%
	3	8446	5393	415	7.7%	7.0%	8.4%
	4	4848	2144	170	7.9%	6.9%	9.1%
	5	1873	0	0	.	.	.
70-79	0	22518	22518	4184	18.6%	18.1%	19.1%
	1	18334	14060	1571	11.2%	10.7%	11.7%
	2	12322	8903	952	10.7%	10.1%	11.4%
	3	7768	4868	505	10.4%	9.5%	11.3%
	4	4251	1935	202	10.4%	9.2%	11.9%
	5	1631	0	0	.	.	.
>=80	0	12455	12455	2958	23.7%	23.0%	24.5%
	1	9497	7244	1071	14.8%	14.0%	15.6%
	2	6082	4275	650	15.2%	14.2%	16.3%
	3	3551	2136	364	17.0%	15.5%	18.7%
	4	1721	802	130	16.2%	13.8%	18.9%
	5	644	0	0	.	.	.
**Past Medical history**							
No	0	80461	80461	9497	11.8%	11.6%	12.0%
	1	70964	54811	3916	7.1%	6.9%	7.4%
	2	50189	36407	2481	6.8%	6.6%	7.1%
	3	33271	21426	1313	6.1%	5.8%	6.5%
	4	19561	8807	561	6.4%	5.9%	6.9%
	5	7809	0	0	.	.	.
1	0	11097	11097	2999	27.0%	26.2%	27.9%
	1	8098	6333	957	15.1%	14.3%	16.0%
	2	5315	3805	466	12.2%	11.2%	13.3%

© 2019 Shankar-Hari M et al. *JAMA Network Open.*

	3	3274	2056	262	12.7%	11.4%	14.3%
	4	1749	769	79	10.3%	8.3%	12.6%
	5	661	0	0	.	.	.
2 or more	0	3190	3190	1323	41.5%	39.8%	43.2%
	1	1867	1393	290	20.8%	18.8%	23.0%
	2	1086	788	110	14.0%	11.7%	16.6%
	3	666	411	53	12.9%	10.0%	16.5%
	4	348	146	22	15.1%	10.2%	21.8%
	5	116	0	0	.	.	.
**APACHE II physiology score quartile**							
Quartile-1	0	29774	29774	3482	11.7%	11.3%	12.1%
	1	26292	20044	1418	7.1%	6.7%	7.4%
	2	18350	13150	906	6.9%	6.5%	7.3%
	3	11993	7615	457	6.0%	5.5%	6.6%
	4	6993	3220	189	5.9%	5.1%	6.7%
	5	2864	0	0	.	.	.
Quartile-2	0	23047	23047	3380	14.7%	14.2%	15.1%
	1	19667	15191	1263	8.3%	7.9%	8.8%
	2	13736	9960	742	7.4%	7.0%	8.0%
	3	9050	5816	386	6.6%	6.0%	7.3%
	4	5274	2368	157	6.6%	5.7%	7.7%
	5	2099	0	0	.	.	.
Quartile-3	0	18704	18704	3011	16.1%	15.6%	16.6%
	1	15693	12174	1073	8.8%	8.3%	9.3%
	2	10961	7905	605	7.7%	7.1%	8.3%
	3	7152	4617	352	7.6%	6.9%	8.4%
	4	4140	1812	143	7.9%	6.7%	9.2%
	5	1587	0	0	.	.	.
Quartile-4	0	23223	23223	3946	17.0%	16.5%	17.5%
	1	19277	15128	1409	9.3%	8.9%	9.8%
	2	13543	9985	804	8.1%	7.5%	8.6%
	3	9016	5845	433	7.4%	6.8%	8.1%
	4	5251	2322	173	7.5%	6.5%	8.6%
	5	2036	0	0	.	.	.
**Site of infection**							
Cardiovascular	0	1612	1612	310	19.2%	17.4%	21.2%
	1	1302	1015	80	7.9%	6.4%	9.7%
	2	926	659	45	6.8%	5.1%	9.0%
	3	609	363	27	7.4%	5.2%	10.6%
	4	321	145	12	8.3%	4.8%	13.9%
	5	123	0	0	.	.	.
Gastrointestinal	0	28630	28630	3561	12.4%	12.1%	12.8%
	1	25069	19449	1364	7.0%	6.7%	7.4%
	2	17839	13065	863	6.6%	6.2%	7.0%
	3	11937	7553	463	6.1%	5.6%	6.7%
	4	6883	3199	207	6.5%	5.7%	7.4%
	5	2845	0	0	.	.	.
Genitourinary	0	6747	6747	972	14.4%	13.6%	15.3%
	1	5775	4286	327	7.6%	6.9%	8.5%
	2	3912	2738	210	7.7%	6.7%	8.7%
	3	2493	1473	112	7.6%	6.4%	9.1%
	4	1329	634	34	5.4%	3.9%	7.4%
	5	571	0	0	.	.	.
Musculoskeletal Dermatological /Haematological	0	5075	5075	674	13.3%	12.4%	14.2%
	1	4401	3323	228	6.9%	6.1%	7.8%
	2	3060	2149	137	6.4%	5.4%	7.5%
	3	1972	1231	81	6.6%	5.3%	8.1%
	4	1118	467	32	6.9%	4.9%	9.5%
	5	418	0	0	.	.	.
Neurological	0	3206	3206	229	7.1%	6.3%	8.1%
	1	2977	2323	94	4.0%	3.3%	4.9%
	2	2198	1571	41	2.6%	1.9%	3.5%
	3	1507	954	26	2.7%	1.9%	4.0%

© 2019 Shankar-Hari M et al. *JAMA Network Open.*

	4	895	385	16	4.2%	2.6%	6.6%
	5	338	0	0	.	.	.
Respiratory	0	43858	43858	6931	15.8%	15.5%	16.1%
	1	36927	28739	2757	9.6%	9.3%	9.9%
	2	25596	18568	1575	8.5%	8.1%	8.9%
	3	16670	10998	832	7.6%	7.1%	8.1%
	4	9901	4339	317	7.3%	6.6%	8.1%
	5	3814	0	0	.	.	.
Unknown	0	5620	5620	1142	20.3%	19.3%	21.4%
	1	4478	3402	313	9.2%	8.3%	10.2%
	2	3059	2250	186	8.3%	7.2%	9.5%
	3	2023	1321	87	6.6%	5.4%	8.1%
	4	1211	553	44	8.0%	6.0%	10.5%
	5	477	0	0	.	.	.
**Number of organ dysfunction**							
Organ dysfunction N=1	0	9727	9727	1086	11.2%	10.6%	11.8%
	1	8641	6651	449	6.8%	6.2%	7.4%
	2	6107	4399	255	5.8%	5.1%	6.5%
	3	4068	2646	145	5.5%	4.7%	6.4%
	4	2451	1118	52	4.7%	3.6%	6.0%
	5	1012	0	0	.	.	.
Organ dysfunction N=2	0	26878	26878	3605	13.4%	13.0%	13.8%
	1	23273	17933	1345	7.5%	7.1%	7.9%
	2	16377	11855	838	7.1%	6.6%	7.5%
	3	10773	6856	409	6.0%	5.4%	6.6%
	4	6263	2810	159	5.7%	4.9%	6.6%
	5	2521	0	0	.	.	.
Organ dysfunction N=3	0	31119	31119	4784	15.4%	15.0%	15.8%
	1	26335	20298	1764	8.7%	8.3%	9.1%
	2	18270	13255	1035	7.8%	7.4%	8.3%
	3	11994	7724	565	7.3%	6.8%	7.9%
	4	6968	3154	227	7.2%	6.3%	8.2%
	5	2771	0	0	.	.	.
Organ dysfunction N=4	0	21075	21075	3433	16.3%	15.8%	16.8%
	1	17642	13675	1276	9.3%	8.9%	9.8%
	2	12238	8845	732	8.3%	7.7%	8.9%
	3	7975	5124	387	7.6%	6.9%	8.3%
	4	4599	2040	179	8.8%	7.6%	10.1%
	5	1771	0	0	.	.	.
Organ dysfunction N= 5 or more	0	5949	5949	911	15.3%	14.4%	16.3%
	1	5038	3980	329	8.3%	7.5%	9.2%
	2	3598	2646	197	7.4%	6.5%	8.5%
	3	2401	1543	122	7.9%	6.7%	9.4%
	4	1377	600	45	7.5%	5.7%	9.9%
	5	511	0	0	.	.	.
**Septic Shock Status**							
Absent	0	81472	81472	11955	14.7%	14.4%	14.9%
	1	69517	53656	4451	8.3%	8.1%	8.5%
	2	48518	35133	2601	7.4%	7.1%	7.7%
	3	31890	20499	1402	6.8%	6.5%	7.2%
	4	18582	8410	587	7.0%	6.5%	7.5%
	5	7412	0	0	.	.	.
Present	0	13276	13276	1864	14.0%	13.5%	14.6%
	1	11412	8881	712	8.0%	7.5%	8.6%
	2	8072	5867	456	7.8%	7.1%	8.5%
	3	5321	3394	226	6.7%	5.9%	7.5%
	4	3076	1312	75	5.7%	4.6%	7.1%
	5	1174	0	0	.	.	.

© 2019 Shankar-Hari M et al. *JAMA Network Open.*

eReferences

1. Shankar-Hari M, Ambler M, Mahalingasivam V, Jones A, Rowan K, Rubenfeld GD. Evidence for a causal link between sepsis and long-term mortality: a systematic review of epidemiologic studies. Crit Care. 2016;20(1):101.
2. Ou SM, Chu H, Chao PW, et al. Long-Term Mortality and Major Adverse Cardiovascular Events in Sepsis Survivors. A Nationwide Population-based Study. Am J Respir Crit Care Med. 2016;194(2):209-217.
3. Prescott HC, Osterholzer JJ, Langa KM, Angus DC, Iwashyna TJ. Late mortality after sepsis: propensity matched cohort study. BMJ. 2016;353:i2375.
4. Young JD, Goldfrad C, Rowan K. Development and testing of a hierarchical method to code the reason for admission to intensive care units: the ICNARC Coding Method. Intensive Care National Audit & Research Centre. Br J Anaesth. 2001;87(4):543-548.
5. Shankar-Hari M, Harrison DA, Rubenfeld GD, Rowan K. Epidemiology of sepsis and septic shock in critical care units: comparison between sepsis-2 and sepsis-3 populations using a national critical care database. Br J Anaesth. 2017;119(4):626-636.
6. Dickman PW, Coviello E. Estimating and modeling relative survival. Stata Journal. 2015;15(1):186-215.

© 2019 Shankar-Hari M et al. *JAMA Network Open.*
